# Nonlocal Coherent Denoising of RF Data for Ultrasound Elastography

**DOI:** 10.1155/2018/7979528

**Published:** 2018-06-24

**Authors:** P. Khavari, A. Asif, M. Boily, H. Rivaz

**Affiliations:** ^1^Department of Electrical and Computer Engineering, Concordia University, Montreal, QC, Canada; ^2^Department of Diagnostic Radiology, McGill University, Montreal, QC, Canada

## Abstract

Ultrasound elastography infers mechanical properties of living tissues from ultrasound radiofrequency (RF) data recorded while the tissues are undergoing deformation. A challenging yet critical step in ultrasound elastography is to estimate the tissue displacement (or, equivalently the time delay estimate) fields from pairs of RF data. The RF data are often corrupted with noise, which causes the displacement estimator to fail in many *in vivo* experiments. To address this problem, we present a nonlocal, coherent denoising approach based on Bayesian estimation to reduce the impact of noise. Despite incoherent denoising algorithms that smooth the B-mode images, the proposed denoising algorithm is used to suppress noise while maintaining useful information such as speckle patterns. We refer to the proposed approach as COherent Denoising for Elastography (CODE) and evaluate its performance when CODE is used in conjunction with the two state-of-art elastography algorithms, namely: (i) GLobal Ultrasound Elastography (GLUE) and (ii) Dynamic Programming Analytic Minimization elastography (DPAM). Our results show that CODE substantially improves the strain result of both GLUE and DPAM.

## 1. Introduction

**Figure 1:**
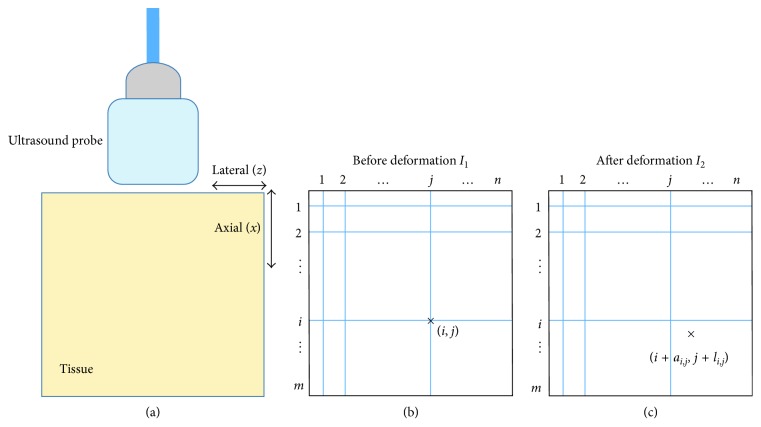
Illustration of ultrasound elastography. (a) A handheld device that induces an external stimulus into the tissues. (b, c) Two successive frames *I*_1_ and *I*_2_ of RF data. The goal of ultrasound elastography was to find the displacement (*a*_*i*,*j*_, *l*_*i*,*j*_) for each pixel (*i*, *j*) in the *I*_1_ RF frame.

Ultrasound elastography determines the viscoelastic properties of tissues and is useful for diagnosis of pathology and for aiding surgeons in the operating room. Broadly speaking, ultrasound elastography can be grouped into two categories [1–6]: dynamic elastography and quasi-static elastography. In this paper, we focus on two state-of-art free-hand palpations and quasi-static elastographic approaches, namely, GLobal Ultrasound Elastography(GLUE) [7] and Real-Time Regularized Ultrasound Elastography (DPAM) [8]. Both approaches use successive pairs of frames of ultrasound RF data to estimate the tissue displacement (also referred to as time delay estimates (TDE)). The derivative of TDE provides an estimate of the induced strain that represents the stiffness or softness of the tissue being imaged.  Figure 1 illustrates the steps involved in quasi-static ultrasound elastography with the handheld device shown on the left handside and the displacement field estimates defined using the two frames on the right.

At the heart of both GLUE and DPAM is an energy minimization approach to determine TDE's. A dynamic programming approach is used in both cases to compute TDE's first at a coarse pixel level. The resolution of the TDE's is then enhanced to the finer subpixel level through analytical minimization. Given that RF ultrasound data can be corrupted by several factors such as thermal and electronic noise, there is a need to compensate for noise in the RF data. Traditional filtering techniques, such as the convolution with a Gaussian kernel, use local continuity in the images to reduce noise. A new class of denoising algorithms, referred to as nonlocal means (NLM) [9], considers data from a much larger “nonlocal” region for denoising. NLM relies on redundancy in images and uses the weighted average of most similar intraframe pixels within a large nonlocal neighbourhood to eliminate noise.

Most NLM-based denoising approaches [10–12] remove noise from processed output of the RF data, which is referred to as B-mode images in ultrasound literature. NLM denoising reduces speckle pattern and generates smooth B-mode images. Ultrasound speckle is useful in several image analysis techniques, such as ultrasound elastography [13, 14], free-hand sensorless 3D ultrasound [15, 16], and quantitative ultrasound [17]. In this work, we focus on ultrasound elastography.

In this paper, we present an alternate approach, wherein the NLM denoising algorithm is applied directly to raw RF data instead of processed B-mode images. We refer to the proposed approach as COherent Denoising for Elastography (CODE) and evaluate its performance on *in vivo* liver ablation data when used in conjunction with two commonly used elastography algorithms, namely: (i) GLobal Ultrasound Elastography (GLUE) [7] and (ii) Dynamic Programming Analytic Minimization elastography (DPAM) [8]. CODE exploits the complete set of information in the RF domain, some of which is likely to be lost in the processing steps used to generate the B-mode images. It is, therefore, our intuition that CODE would result in superior denoising results. Using information in RF data to generate visually informative B-mode images is challenging [18]. To illustrate the superiority of CODE, both mathematical analysis and experimental results are included in the paper. Our comparisons corroborate our intuition and verify the usefulness of CODE.

The rest of this paper is organized as follows. In Section 2, we introduce GLUE and DPAM as representative quasi-static elastography approaches.  Section 3 provides background on nonlocal denoising and introduces CODE as a Bayesian estimator. In Section 4, we explore the ability of CODE on simulation data. Experimental results using phantom and *in vivo* data are included in Section 5. Finally, we conclude the paper in Section 6.

## 2. Quasi-Static Elastography: GLUE and DPAM

Both DPAM and GLUE are quasi-static approaches based on the optimization of a regularized cost function to determine tissue displacements. They both aim at finding the axial and lateral displacements (*a* and *l*) of all samples of RF data as shown in Figure 1. DPAM uses dynamic programming (DP) to first estimate the integer displacement of a *seed-line* in terms of the number of pixels and then applies analytical minimization (AM) to fine tune the estimated displacement to the subpixel level. The strain image is obtained using the spatial differentiation of the displacement field. GLUE also uses DP for estimating the integer tissue displacements and refines the estimates to subpixels for the entire image simultaneously. In other words, GLUE solves an optimization function where both axial and lateral displacements of every sample of the RF frame are unknowns, that is, in the order of a million variables. This is in contrast to DPAM, which refines the estimates line-by-line. The strain image again is calculated based on the differentiation of displacement map similar to DPAM. Although GLUE and DPAM perform well in most cases, they may not converge to the correct solution in the presence of excessive noise. In the next section, we present our denoising approach used to reduce the impact of noise in the RF domain.

## 3. The Nonlocal Denoising Approach

The central idea behind this paper is to apply coherent denoising on RF data. Unlike incoherent denoising approaches that process the B-mode images to remove noise (resulting in spatial averaging and significant loss of speckle patterns), the proposed approach retains speckle patterns. We first outline NLM, which is followed by a description of the CODE algorithm, including an analytical justification of why CODE provides better denoising results.

### 3.1. Nonlocal Means

Let *v*(*i*) be the observed value of the discretized image for pixel *i* and *u*(*i*) be its true value. Due to the presence of noise *n*(*i*), we have

(1) $$\begin{array}{c} v\left(i\right)=u\left(i\right)+n\left(i\right). \end{array}$$

**Figure 2:**
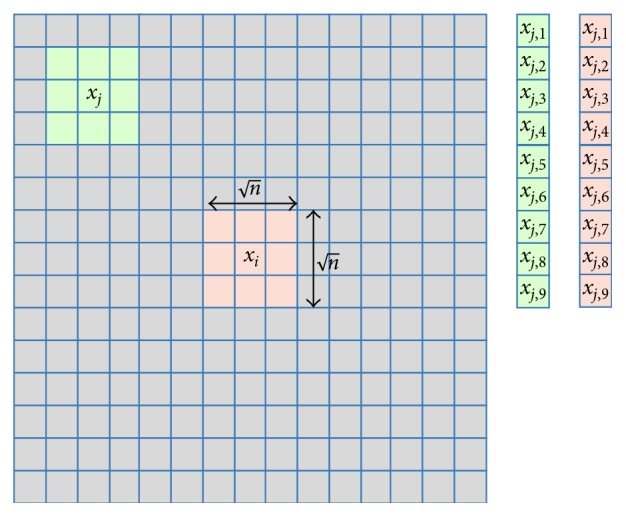
Illustration for the patch and vectorized indices used in the proposed CODE approach for *n*=9.

To simplify our explanation, we focus on 1D signals, but our results are generalizable to 2D images. In fact, the experimental results included in Section 4 are for 2D phantom and in vivo liver ablation data. To denoise the image for each pixel *i*, NLM searches a reference area of the image within a rectangular *search window*Δ_*i*_, which is centered around pixel *i* (Figure 2). A neighbourhood *N*_*i*_ of known dimension is selected around pixel *i* and compared to neighbourhood *N*_*j*_ around pixel *j* for all *j* ∈ Δ_*i*_. For pixel *i*, weight *w*(*i*, *j*) is assigned to each pixel *j*. The value of pixel *i* is then replaced by

(2) $$\begin{array}{c} \text{NLM}\left[v\right]\left(i\right)=\sum_{j\in \Delta_{i}}w\left(i,j\right)\;*\;v\left(j\right). \end{array}$$

The distance metric is proportional to the square of the Euclidian distance between the two patches. The weight is then calculated as

(3) $$\begin{array}{c} w\left(i,j\right)=\frac{1}{Z_{i}}\exp\left\{-\frac{{\left\|v\left(N_{i}\right)-v\left(N_{j}\right)\right\|}_{2,a}^{2}}{h^{2}}\right\}. \end{array}$$

Based on (3), it is clear that the weight is the convolution of a Gaussian with standard deviation *a* > 0 and the squared Euclidean distance between two neighbourhoods ‖*v*(*N*_*i*_) − *v*(*N*_*j*_)‖_2_^2^, for *N*_*i*_ and *N*_*j*_. The smoothing parameter *h* controls the contribution of the Gaussian-Euclidean distance exponent in the weights. The normalization factor *Z*_*i*_ for pixel *i* is given by

(4) $$\begin{array}{c} Z\left(i\right)=\underset{j\in \Delta_{i}}{\sum }\exp\left\{-\frac{{\left\|v\left(N_{i}\right)-v\left(N_{j}\right)\right\|}_{2,a}^{2}}{h^{2}}\right\}, \end{array}$$

where the weight is normalized to ensure that the dynamic range of the NLM[*v*](*i*) is the same as that of its counterpart *v*(*i*).

### 3.2. The Proposed Bayesian CODE Framework

Noise in ultrasound B-mode images originates from piezoelectric sensors and data acquisition card. Depending on the application, the level of noise can even be higher. For example, ablation treatment generates heat and microbubbles that severely deteriorate RF data [19–21]. Both logarithmic compression and envelope detection steps, applied to derive the B-mode image, are nonlinear operations that complicate measurement noise added by sensors and acquisition card. Our CODE approach eliminates noise introduced by sensors and acquisition card before the nonlinear logarithmic compression and envelope detection by applying NLM directly to RF ultrasound data.

We now provide an analytical explanation of why NLM denoising is adapted for the RF domain. Let **g**(**x**) and **o**(**x**) be vectorized ground truth and observed patches of size *n* centered at pixel *x*_*i*_ of RF data (Figure 2). We define them as **g**(**x**)=*g*(*x*_*k*_) with *x*_*k*_ ∈ *N*_*g*_(*x*) and **o**(**x**)=*o*(*x*_*k*_), where *x*_*k*_ ∈ *N*_*o*_(*x*) and {*N*_*o*_, *N*_*g*_} are the neighbourhoods (patches) of size $\left(\sqrt{n}\times \sqrt{n}\right)$ around the central pixel *x* in ground truth and observed images. Our goal is to derive the Bayesian estimator $\hat{g}\left(x\right)$ for patch **g**(**x**) based on the observed patch **o**(**x**). Defining the optimal estimator by minimizing the posterior expected loss as

(5) $$\begin{array}{c} \text{E}\left[L\left(g\left(x\right),\hat{g}\left(x\right)\right)\right]=\sum_{g\left(x\right)\in \Gamma }\left[L\left(g\left(x\right),\hat{g}\left(x\right)\right)\right]p\left(\left.g\left(x\right)\right|o\left(x\right)\right), \end{array}$$

where Γ constitutes all possible outcomes of **g**(**x**), the loss function is given by

(6) $$\begin{array}{c} L\left(g\left(x\right),\hat{g}\left(x\right)\right)={\left\|g\left(x\right)-\hat{g}\left(x\right)\right\|}^{2}. \end{array}$$

Substituting (6) in (5), the optimal Bayesian estimator is

(7) $$\begin{array}{c} \hat{g}{\left(x\right)}_{\text{opt}}=\underset{\hat{g}\left(x\right)}{\arg\;\min}\sum_{g\left(x\right)}{\left\|g\left(x\right)-\hat{g}\left(x\right)\right\|}^{2}p\left(\left.g\left(x\right)\right|o\left(x\right)\right)\\ \;\;\;=\sum_{g\left(x\right)}g\left(x\right)p\left(\left.g\left(x\right)\right|o\left(x\right)\right). \end{array}$$

Equation (7) can be expressed as

(8) $$\begin{array}{c} \hat{g}{\left(x\right)}_{\text{opt}}=\sum_{g\left(x\right)}g\left(x\right)\frac{p\left(g\left(x\right),o\left(x\right)\right)}{p\left(o\left(x\right)\right)}=\frac{\sum_{g\left(x\right)}g\left(x\right)p\left(\left.o\left(x\right)\right|g\left(x\right)\right)p\left(g\left(x\right)\right)}{\sum_{g\left(x\right)}p\left(\left.o\left(x\right)\right|g\left(x\right)\right)p\left(g\left(x\right)\right)}. \end{array}$$

Only a subset of Γ is accessible in the search region of the central pixel *x*_*i*_. We refer to this subset as the search region, SR(**x**)={**g**_1_(*x*), **g**_2_(*x*), **g**_3_(*x*),…, **g**_*K*_(*x*)}. Assuming that cardinality of SR is *K* and *p*(**g**(**x**)) is uniformly distributed, that is, *p*(**g**_*i*_(*x*))=1/*K*, for  all(0 ≤ *i* ≤ *K*), (8) simplifies to

(9) $$\begin{array}{c} \hat{g}\left(x_{i}\right)=\frac{\sum_{j=1}^{K}g\left(x_{j}\right)p\left(\left.o\left(x_{i}\right)\right|g\left(x_{j}\right)\right)}{\sum_{j=1}^{K}p\left(\left.o\left(x_{i}\right)\right|g\left(x_{j}\right)\right)}, \end{array}$$

where $\hat{g}\left(x_{i}\right)$ is the optimal estimator based on the uniform distribution assumption. Given the ground truth is not accessible, we substitute the observed value of the neighbourhood patches to get

(10) $$\begin{array}{c} \hat{g}\left(x_{i}\right)=\frac{\sum_{j=1}^{K}o\left(x_{j}\right)p\left(\left.o\left(x_{i}\right)\right|o\left(x_{j}\right)\right)}{\sum_{j=1}^{K}p\left(\left.o\left(x_{i}\right)\right|o\left(x_{j}\right)\right)}. \end{array}$$

Given that the noise in the RF data is modelled as an additive Gaussian noise [22, 23], we have

(11) $$\begin{array}{c} o\left(x\right)=g\left(x\right)+v\left(x\right), \end{array}$$

where **v**(**x**) is the additive white Gaussian noise with variance *σ*^2^. By assuming that the likelihood can be factorized as

(12) $$\begin{array}{c} p\left(\left.o\left(x_{i}\right)\right|o\left(x_{j}\right)\right)=\prod_{k=1}^{n}p\left(\left.o\left(x_{i,k}\right)\right|o\left(x_{j,k}\right)\right), \end{array}$$

where *x*_*i*,*k*_ ∈ *N*(*x*_*i*_) and *x*_*j*,*k*_ ∈ *N*(*x*_*j*_) are the counterpart pixels in the patches with central pixels *x*_*i*_ and *x*_*j*_. Therefore, *p*(**o**(*x*_*i*_)|**o**(*x*_*j*_)) is multivariant normal distributed *p*(**o**(*x*_*i*_)|**o**(*x*_*j*_)) ~ *𝒩*(**o**(*x*_*j*_), *σ*^2^**I**_*n*_). Notation **I**_*n*_ is the identity matrix. Thus, the filter in (10) can be adapted to remove the noise of RF data as

(13) $$\begin{array}{c} \hat{g}\left(x_{i}\right)=\frac{1}{C\left(x_{i}\right)}\sum_{j=1}^{K}\exp^{-\;\left({\left\|o\left(x_{i}\right)-o\left(x_{j}\right)\right\|}^{2}/h^{2}\right)}o\left(x_{j}\right),\\ \text{with }C\left(x_{i}\right)=\sum_{j=1}^{K}\exp^{-\;\left({\left\|o\left(x_{i}\right)-o\left(x_{j}\right)\right\|}^{2}/h^{2}\right)}. \end{array}$$

Equation (13) is also known as the NLM algorithm. By considering the normal distributed assumption, (13) can be adapted for denoising the RF data by replacing *h*^2^=2*σ*^2^. Therefore, the adapted filter for denoising the RF data (CODE) is

(14) $$\begin{array}{c} \hat{g}\left(x_{i}\right)=\frac{1}{C\left(x_{i}\right)}\sum_{j=1}^{K}\exp^{-\;\left({\left\|o\left(x_{i}\right)-o\left(x_{j}\right)\right\|}^{2}/2\sigma^{2}\right)}o\left(x_{j}\right),\\ \text{with }C\left(x_{i}\right)=\sum_{j=1}^{K}\exp^{-\;\left({\left\|o\left(x_{i}\right)-o\left(x_{j}\right)\right\|}^{2}/2\sigma^{2}\right)}. \end{array}$$

This filter is based on the noise statistics of RF data. CODE is, therefore, the optimal denoising approach for removing noise in (11) within the RF domain.

Kevrann et al. [12] and Coupe et al. [10] have developed similar Bayesian estimators but for reducing the speckles pattern in the B-mode image. Aligned with the mathematical Bayesian estimator, the properties of noise in RF data show the usefulness of CODE for removing noise from the RF ultrasound data.

## 4. Simulation Validation for Code

To assess the performance of the CODE approach, the Field II [24] software is used to simulate RF data from a lesion phantom of size 60, 50, and 10 mm in axial, lateral, and out-of-plane directions, respectively. The phantoms consist of two classes of background and target tissues. To determine the precision and sensitivity of the CODE, three different setups with 5, 10, and 15 scatterers per resolution cell distributed randomly within the phantom are used. Different realizations for each group of scatterers are generated. The RF output of Field II is corrupted by adding additive white Gaussian noise with a SNR of 5 dB.

**Figure 3:**
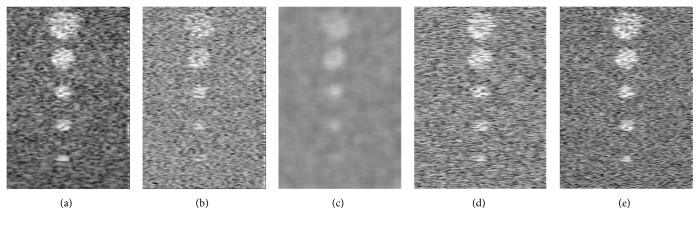
Field II simulation results. The noisy input has substantially less contrast than the ground truth image. NLM is designed to remove speckle and therefore substantially reduces image detail. CODE output is closest to the ground truth. (a) Ground truth, (b) noisy, (c) NLM, (d) Gaussian on RF, and (e) CODE.

**Figure 4:**
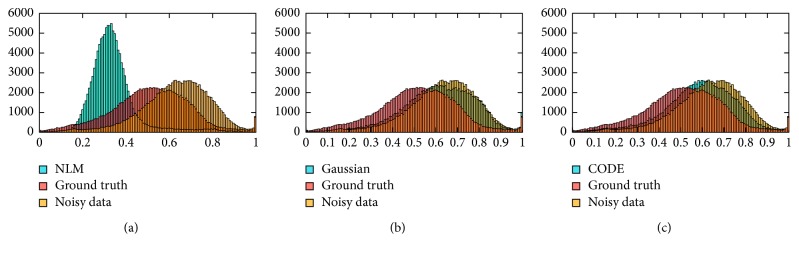
(a) Histograms of NLM, ground truth, and noisy data. (b) Histograms of Gaussian denoising, ground truth, and noisy data. Finally, (c) is the same as (b) except the histogram of NLM replaced by that of CODE.

Figure 3 shows the results of NLM applied to B-mode images. As expected, NLM performs incoherent averaging and removes speckle pattern. This is desired for many applications such as segmentation and registration [25], but not in elastography. Figure 3 also shows the results of applying a Gaussian kernel to the RF data. Since averaging is performed in the RF domain, the speckle pattern is retained. Finally, the results of CODE denoising are also shown in this figure, which visually outperforms other methods in terms of similarity to the original B-mode image.  Figure 4 compares the histogram of the B-mode of these three images. Since the distribution of noise-free image (ground truth) is known, we used the following chi-square test as a quantitative parameter for comparison:

(15) $$\begin{array}{c} \chi^{2}=\sum_{t=1}^{m}\frac{{\left(O_{t}-E_{t}\right)}^{2}}{E_{t}}, \end{array}$$

where *O*_*t*_ is the observed value, *E*_*t*_ is the expected value, and *m* is the number of bins (256 bins of grey levels for simulated images). The chi-squared criterion for distribution and sum of squared difference (SSD) between original and filtered images using NLM, Gaussian with kernel width of 5 and smoothing parameter 1, and CODE with search region 21, kernel width 5, and smoothing parameter 5, are compared in Table 1. In both cases (chi-squared and SSD), CODE outperforms the conventional NLM approach and Gaussian denoising applied directly on RF data, as demonstrated in theory in Section 3.2.

Moreover, with respect to simulations in Field II, the ground truth is available to study error variance of all 3 distributions of scatterers. The error variance is measured using normalized root mean square error (NRMSE) defined as

(16) $$\begin{array}{c} \text{NRMSE}\left(G,I_{d}\right)=\frac{\sqrt{\left(\sum_{i=1}^{n}\sum_{j=1}^{m}{\left(I_{d}\left(i,j\right)-G\left(i,j\right)\right)}^{2}\right)/\left(m\;*\;n\right)}}{\max\left(G\right)-\min\left(G\right)}, \end{array}$$

where *G* is ground truth of Field II, *I*_*d*_ is either noisy image or denoised version using NLM or Gaussian denoising.  Table 2 shows that the error variance for the CODE method is minimum in comparison with other denoising.

## 5. Phantom and *In Vivo* Elastography

**Figure 5:**
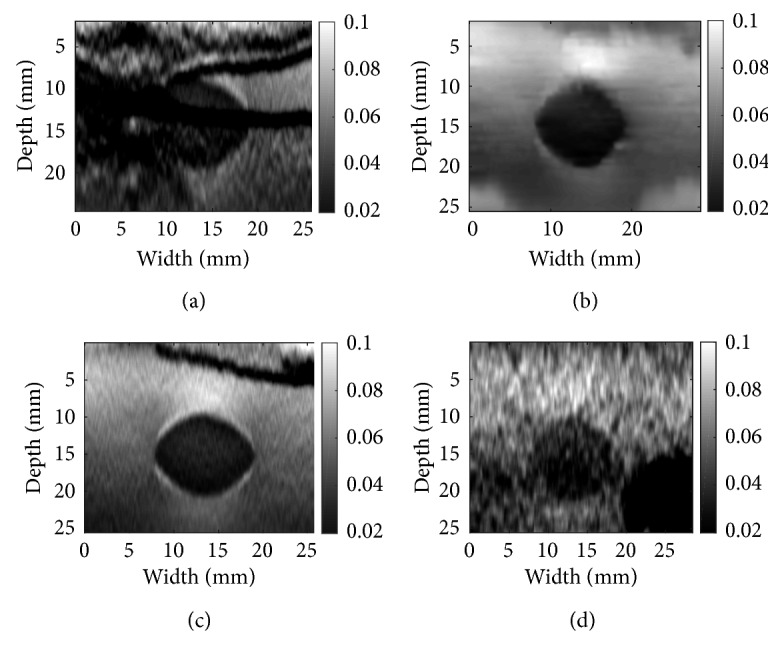
Denoising results for phantom data: (a) DPAM alone; (b) DPAM with CODE; (c) DPAM with Gaussian; (d) DPAM with NLM. For CODE, the dimension of the search window is (11 × 11), size of the neighbourhood is (3 × 3), and the smoothing parameter *h* is set to 11. For (c), the kernel size is (3 × 3) and smoothing parameter is 1. For (d), the NLM properties are set as (b), but they are applied on B-mode.

**Figure 6:**
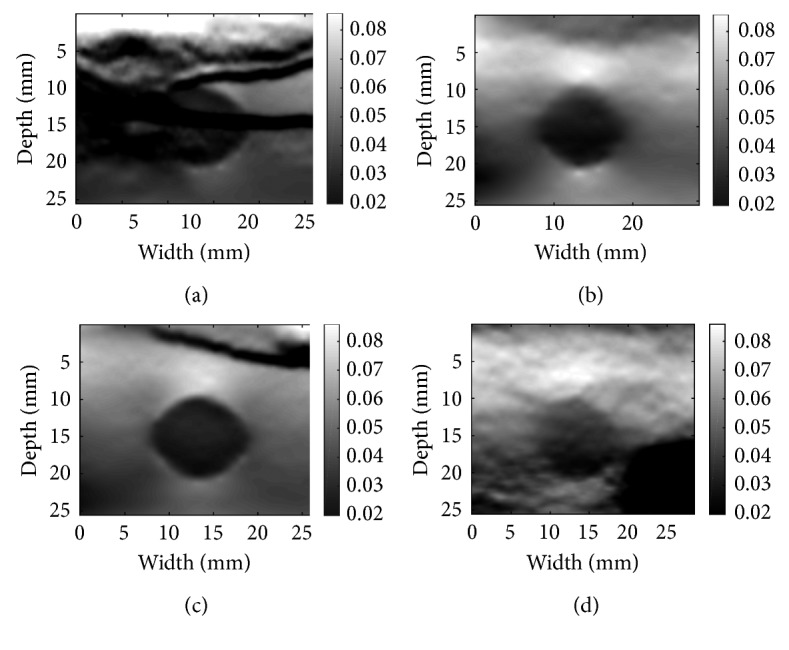
Denoising results for phantom data: (a) GLUE alone; (b) GLUE with CODE; (c) GLUE with Gaussian; (d) GLUE with NLM. For CODE, the dimension of the search window is (11 × 11), size of the neighbourhood is (3 × 3), and the smoothing parameter *h* is set to 11. For (c), the kernel size is (3 × 3) and smoothing parameter is 1. For (d), the NLM properties are set as (b), but it is applied on B-mode.

**Figure 7:**
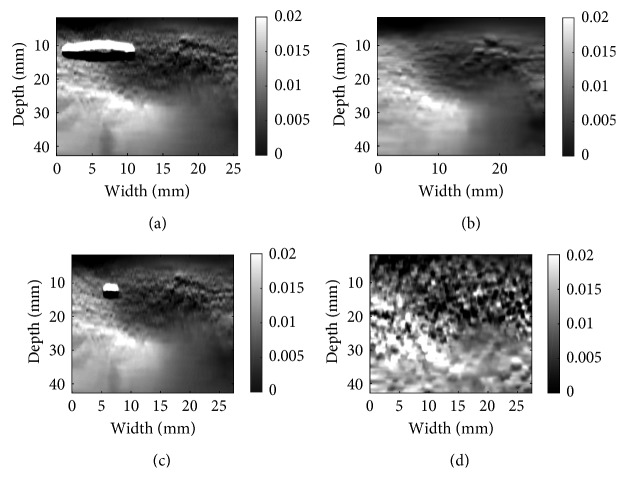
Same as Figure 5 except in vivo liver ablation, patient data are used: (a) DPAM alone; (b) DPAM with CODE; (c) DPAM with Gaussian; (d) DPAM with NLM.

**Figure 8:**
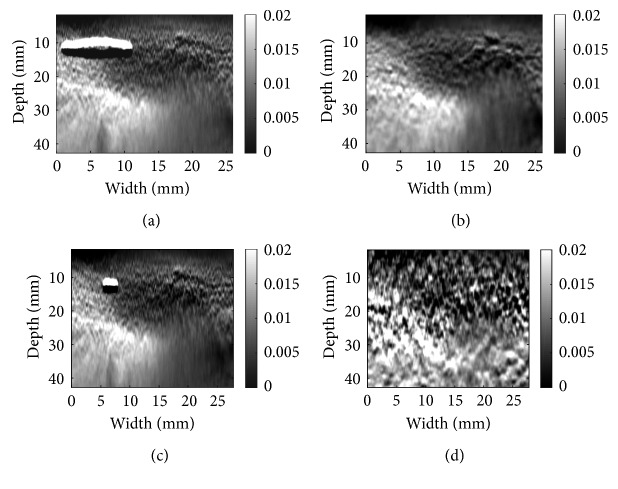
Same as Figure 6 except in vivo liver ablation, patient data are used: (a) GLUE alone; (b) GLUE with CODE; (c) DPAM alone; (d) DPAM with CODE.

**Figure 9:**
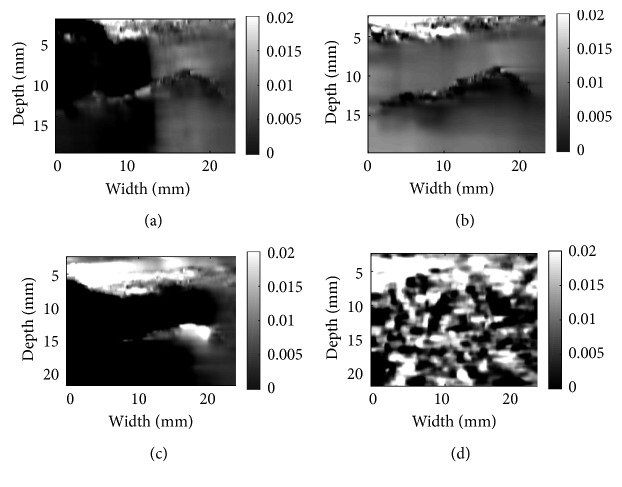
Same as Figure 5 except in vivo liver ablation, patient tendon data are used: (a) DPAM alone; (b) DPAM with CODE; (c) DPAM with Gaussian; (d) DPAM with NLM.

**Figure 10:**
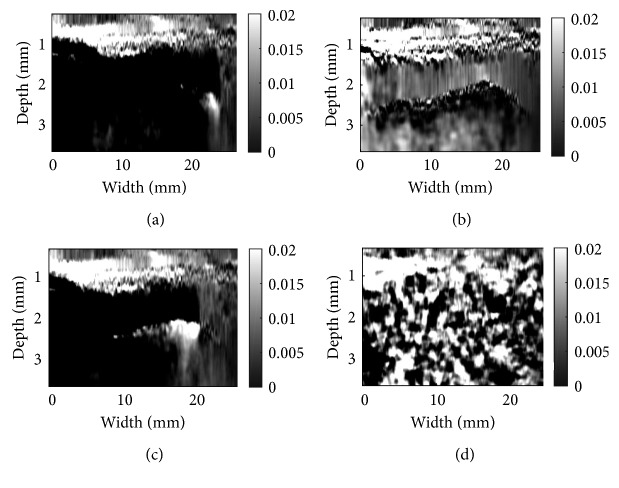
Same as Figure 6 except in vivo liver ablation, patient tendon data are used: (a) GLUE alone; (b) GLUE with CODE; (c) GLUE with Gaussian; (d) GLUE with NLM.

We study 3 different cases of phantom data, *in vivo* liver ablation data, and tendon data for both GLUE and DPAM. The results are provided in Figures 5–10. The window size of 3 provides correct strain map, for CODE meanwhile requires the minimum computational budget. To be fair in comparison, the window size is the same for both NLM and Gaussian denoising.

Phantom data in Figures 5 and 6 are obtained from a CIRS breast phantom (Norfolk, VA) under free-hand palpation. There is excessive out-of-plane motion between the two processed images, and therefore, the DP step fails. This leads to failure in both DPAM and GLUE, which is apparent as black horizontal artifacts in (a), (c) and black artifact at right down corner of (d) for both mentioned figures. However, CODE removes the noise from the RF data and leads to a strain image with low noise and high contrast. The phantom contains a cyst in the middle with certain elasticity surrounded by another tissue. Those artifacts as described are failing to depict the tissue around the cyst or the cyst elasticity by showing different elasticities.

Patient data in Figures 7 and 8 were acquired from a patient undergoing open-surgical radiofrequency thermal ablation for primary or secondary liver cancer. These data are available online [8]. The Institutional Ethical Review Board at Johns Hopkins University approved all experimental procedures involving human subjects. For the patient data, ablation procedure generates substantial amount of noise in the RF data [19–21]. As a result of excessive noise, DP fails, which generates the horizontal black and white bands in the top left of (a), (c), and (d). Although the environment is extremely noisy, the well-adapted CODE method denoises the RF data in a way that both algorithms are able to get the correct strain map for patient data. The ablation operation coagulates the tissue, which makes the tissue stiffer. The coagulated tissue is often referred to as ablation lesion, and its size should be bigger than the tumor to ensure that the entire tumor is ablated. The strain images in Figures 7(b) and 8(b) clearly show the ablation lesion as a dark region with low strain (i.e., hard). CODE helps to remove noise in RF data, which leads to less noisy strain images. Such strain images can help the surgeon to minimize the cancer recurrence rate. However, NLM and Gaussian fail to reconstruct the strain map and show sudden changes in tissue that are misleading and violate tissue continuity.

We also evaluate CODE on data collected from patellar tendon. These data were collected at the PERFORM Centre at Concordia University. Ethics approval was obtained for this study from Quebec's Ministere de la Sante et des Services Sociaux, and all subjects signed a consent form to participate. Data are collected using an Alpinion ECube ultrasound machine (Bothell, WA) with a L3-12 linear transducer at the centre frequency of 11 MHz with sampling frequency of 40 MHz. The results are shown in Figures 9 and 10. The probe is held stationary, and the subject flexes his knee joint during data collection. CODE removes the noise in the RF data and results in a more meaningful strain image.

## 6. Conclusions

In this paper, we have proposed a denoising algorithm, referred to as the CODE (COherent Denoising for Elastography) approach for ultrasound elastography. CODE is applied directly to the RF data and has the ability to eliminate noise, while retaining relevant speckle patterns. This is demonstrated using phantom and experiments based on *in vivo* clinical data. The results of CODE are used for GLUE and DPAM, which verifies the effectiveness of the proposed CODE. More clinical studies are needed to fully verify the benefits of the CODE algorithm.

## Figures and Tables

**Table 1:** Values of chi-square and SSD for reconstructed images..

Scatterer	Chi^2^			SSD		
	NLM	Gaussian	CODE	NLM	Gaussian	CODE
5/mm^3^	9702.33	167.78	**95.49**	76250.03	2240.55	**2119.90**
10/mm^3^	86361.68	253.65	**60.18**	7482.55	2242.51	**1909.44**
15/mm^3^	6108.31	294.90	**27.63**	7961.00	2300.42	**1536.20**

The ground truth was obtained from a Field II simulation

**Table 2:** Using Field II ground truth for evaluation of NRMSE for different denoising and noisy images.

Method	5/mm^3^	10/mm^3^	15/mm^3^
Noisy	0.1501	0.1298	0.1284
NLM	0.3210	0.3208	0.3182
Gaussian	0.1595	0.1478	0.1442
CODE	**0.1354**	**0.1216**	**0.1203**

## Data Availability

The data collected for this publication cannot be shared online due to the requirements of the ethics approval and other constraints. Some of the data used in this paper are available online [8].
